# Solid Organ Transplantation in Patients with Inflammatory Bowel Diseases (IBD): Analysis of Transplantation Outcome and IBD Activity in a Large Single Center Cohort

**DOI:** 10.1371/journal.pone.0135807

**Published:** 2015-08-19

**Authors:** Fabian Schnitzler, Matthias Friedrich, Johannes Stallhofer, Ulf Schönermarck, Michael Fischereder, Antje Habicht, Nazanin Karbalai, Christiane Wolf, Marianne Angelberger, Torsten Olszak, Florian Beigel, Cornelia Tillack, Burkhard Göke, Reinhart Zachoval, Gerald Denk, Markus Guba, Christian Rust, Norbert Grüner, Stephan Brand

**Affiliations:** 1 Department of Medicine II—Grosshadern, Ludwig-Maximilians-University (LMU), Munich, Germany; 2 Department of Preventive Dentistry and Periodontology, Ludwig-Maximilians-University (LMU), Munich, Germany; 3 Department of Medicine IV Nephrology—Grosshadern, Ludwig-Maximilians-University (LMU), Munich, Germany; 4 Transplantation Center—Grosshadern, Ludwig-Maximilians-University (LMU), Munich, Germany; 5 Max-Planck-Institute of Psychiatry, Munich, Germany; 6 Department of Surgery—Grosshadern, Ludwig-Maximilians-University (LMU), Munich, Germany; 7 Department of Gastroenterology, Hospital Barmherzige Brüder, Munich, Germany; Charité, Campus Benjamin Franklin, GERMANY

## Abstract

**Background:**

Currently, limited data of the outcome of inflammatory bowel disease (IBD) in patients after solid organ transplantation (SOT) are available. We aimed to analyze effects of SOT on the IBD course in a large IBD patient cohort.

**Methods:**

Clinical data from 1537 IBD patients were analyzed for patients who underwent SOT (n = 31) between July 2002 and May 2014. Sub-analyses included SOT outcome parameters, IBD activity before and after SOT, and efficacy of IBD treatment.

**Results:**

4.74% of patients with ulcerative colitis (UC) and 0.84% of patients with Crohn’s disease (CD) underwent SOT (p = 2.69 x 10^−6^, UC vs. CD). 77.4% of patients with SOT underwent liver transplantation (LTx) with tacrolimus-based immunosuppressive therapy after SOT. All LTx were due to primary sclerosing cholangitis (PSC) or PSC overlap syndromes. Six patients (19.4%) required renal transplantation and one patient (3.2%) heart transplantation. A survival rate of 83.9% after a median follow-up period of 103 months was observed. Before SOT, 65.0% of patients were in clinical remission and 5 patients received immunosuppressive therapy (16.1%). After SOT, 61.0% of patients were in remission (p = 1.00 vs. before SOT) and 29.0% required IBD-specific immunosuppressive or anti-TNF therapy (p = 0.54 vs. before SOT). 42.9% of patients with worsening of IBD after SOT were at higher risk of needing steroid therapy for increased IBD activity (p = 0.03; relative risk (RR): 10.29; 95% CI 1.26–84.06). Four patients (13.0%) needed anti-TNF therapy after SOT (response rate 75%).

**Conclusions:**

SOT was more common in UC patients due to the higher prevalence of PSC-related liver cirrhosis in UC. Despite mainly tacrolimus-based immunosuppressive regimens, outcome of SOT and IBD was excellent in this cohort. In this SOT cohort, concomitant immunosuppressive therapy due to IBD was well tolerated.

## Introduction

The clinical course of inflammatory bowel diseases (IBD) such as ulcerative colitis (UC) and Crohn’s disease (CD) is typically characterized by alternating episodes of flares and remission. In up to one third of IBD patients, extraintestinal manifestations such as primary sclerosing cholangitis (PSC) or renal dysfunction (e.g., due to amyloidosis) are found [1–3].

PSC is a chronic cholestatic liver disease with chronic inflammation and fibrosis of hepatic bile ducts, resulting in liver cirrhosis and progressive impairment of liver function and consecutive liver failure in a subgroup of PSC patients [3, 4]. Liver transplantation is currently the only curative therapy for PSC as medical treatments are limited and non-curative in PSC [5]. PSC is more frequent in UC patients than in CD patients with prevalence rates of PSC ranging from 0.76% to 5.4% in UC patients and from 1.2% to 3.4% in CD patients [1, 6–8]. Most IBD patients with PSC display a characteristic disease course compared to IBD patients without cholestatic liver diseases [4, 8–17]. Furthermore, the frequency of pancolitis is higher in UC-PSC patients with more right-sided colitis; and more of these patients have rectal sparing and backwash ileitis, although the course of UC is often mild [4, 9–12, 14, 15, 18]. In contrast, the risk of malignancies including colorectal cancer (CRC) and cholangiocarcinoma is significantly increased in UC patients with concomitant PSC, independently from the underlying risk of CRC in UC alone [13, 16, 17, 19]. In addition, the risk of pouchitis was reported to be high after proctocolectomy with ileal pouch-anal anastomosis (IPAA) [9].

Given the high prevalence of PSC among IBD patients, PSC is the most frequent cause for liver transplantation (LTx) in IBD patients. Another less frequent cause for solid organ transplantation (SOT) in IBD patients is renal insufficiency, e.g., due to amyloidosis [2, 20]. In IBD patients undergoing SOT, the disease course is highly variable after SOT and data on the subsequent IBD course after SOT are conflicting [2, 3, 9–18, 20–31]. A recently published meta-analysis included a total of 609 IBD patients of 14 clinical studies and investigated the natural history of IBD after LTx in patients with PSC/UC. Among these IBD patients, one third (31%) showed improvement of IBD activity after LTx, 39% of patients displayed no significant change of IBD activity, whereas in 30% of patients the IBD activity worsened after LTx with need for treatment intensification after LTx [5]. Similarly, after renal transplantation, approximately 30% of patients develop IBD flares and one fifth of patients have to undergo colectomy after renal transplantation [32–35]. Therefore, for approximately one third of IBD patients treatment has to be adapted due to the increasing activity of IBD after SOT.

Anti-tumour necrosis factor alpha (TNF-α) therapy has proven to be an effective therapeutic option in patients with refractory IBD in numerous clinical trials. Therefore, anti-TNF-α therapy represents a treatment option in IBD patients who underwent SOT. However, clinical experience of anti-TNF-α therapy in IBD patients after SOT is very limited. To date, a total of 21 IBD patients including patients with UC, CD, indeterminate colitis and pouchitis, have been treated with infliximab or adalimumab after LTx [36–40]. Some case reports were published on anti-TNF-α therapy in IBD patients after renal transplantation but no data exist on anti-TNF-α therapy in IBD after heart transplantation [41, 42].

Given the rare incidence of SOT in IBD patients, our large IBD patient cohort enabled us to perform a large single center study (n = 31 SOT cases) on the IBD disease course and anti-TNF-treatment efficacy before and after SOT in a well-characterized IBD cohort.

One aim was to investigate the outcome of SOT in IBD patients and to evaluate the course of IBD before and after SOT. In addition, we aimed to analyze the treatment outcome of anti-TNF therapy among these patients. These data were finally compared to other available clinical trials and analyses of SOT in IBD patients.

## Materials and Methods

### Ethical Statement

All individuals gave their written, informed consent prior to study inclusion. The study was approved by the local Ethics committee (Ludwig-Maximilians-University Munich) and adhered to the ethical principles for medical research involving human subjects of the Helsinki Declaration.

### Study population

All IBD patients were recruited from the IBD outpatient department of the University Hospital Munich-Grosshadern and from our Center for Solid Organ Transplantation (Ludwig-Maximilians-University Munich, Germany). Databases of all IBD patients who were followed at the IBD outpatient department and of all patients who underwent SOT at the University Hospital Munich-Grosshadern or were followed after SOT at our Center for Solid Organ Transplantation, respectively, were merged to identify IBD patients who underwent SOT. Two senior gastroenterologists viewed relevant data of the 31 IBD patients who underwent SOT between July 2002 and May 2014. Clinical data was collected prospectively. However, data analysis was performed retrospectively. Two senior gastroenterologists analyzed the data which were recorded by patients’ chart analysis and a detailed questionnaire based on an interview at time of enrolment. All patients were regularly seen at the IBD outpatient department and at the Center for Solid Organ Transplantation at the University Hospital Munich—Grosshadern. The diagnosis of UC and CD was based on the Montréal classification including endoscopic, radiological, and histopathological parameters [43]. IBD activity was evaluated clinically before and after SOT and was based on endoscopic findings before and after SOT. Endoscopic assessment for UC was based on the Mayo endoscopic subscore with (0) for inactive disease, (1) for mild disease with erythema, decreased vascular pattern, mild friability, (2) for moderate disease with marked erythema, absent vascular pattern, friability, erosions and (3) for severe disease with spontaneous bleeding and ulcerations. For CD, endoscopic activity was defined as “remission”in case of the absence of erosions, ulcers and stenosis and fistulas, respectively and “mild”in case of signs of inflammation with erosions and absence of ulcers, stenosis and fistulas, respectively, and “severe”in case of ulcerations, stenosis or fistulas. For clinical assessment of CD, the Crohn's Disease Activity Index (CDAI) was used; a score of < 150 points was defined as clinical remission. For UC, the Clinical Activity Index (CAI, Lichtiger score) was used; a CAI of ≤ 4 points was defined as clinical remission. For endoscopic activity, the last endoscopy before SOT and the first endoscopy after SOT were analyzed. Steroid treatment after SOT for IBD was defined as daily steroid treatment > 10 mg prednisolone due to high IBD activity.

### Statistical analysis

Data were described with proportions for categorical variables and median with range for continuous variables. Crude associations between categorical variables were assessed with the Chi-square test or the Fisher’s exact test, where appropriate. Quantitative variables were compared between subgroups using Student’s t-test. All tests were two-tailed and p-values < 0.05 were considered as significant.

## Results

### Solid organ transplantation in IBD patients

**Fig 1:**
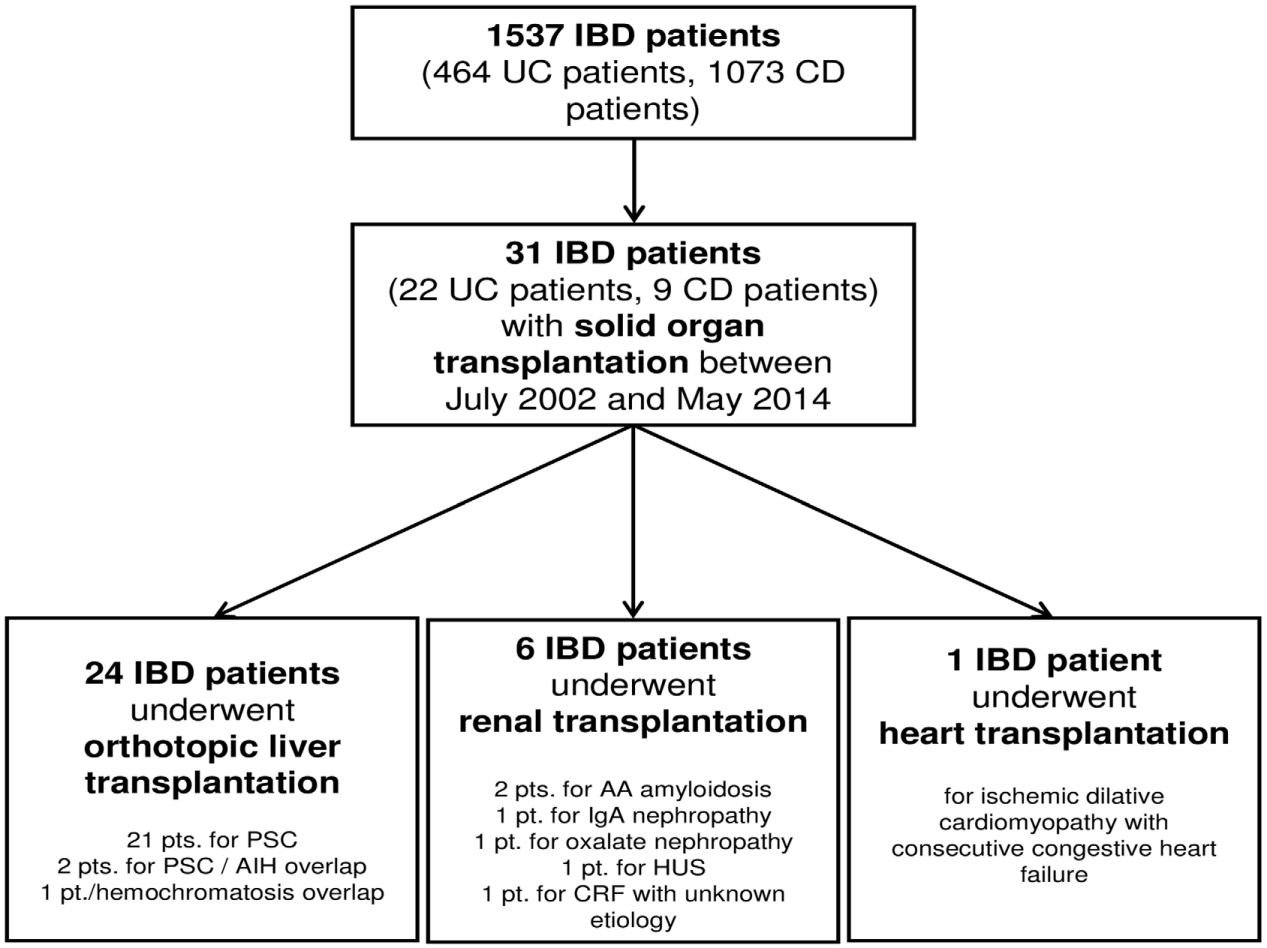
Indication for solid organ transplantation (SOT). A total of 31 patients (22 UC patients and 9 CD patients underwent SOT between July 2002 and May 2014). Twenty-four IBD patients (21 UC patients and 3 CD patients) underwent orthotopic LTx (77.4%) and 6 IBD patients (1 UC patient and 5 CD patients) underwent renal transplantation (19.4% off all SOT) and one CD patient underwent heart transplantation (3.2% of all SOT) (PSC, primary sclerosing cholangitis; AIH, autoimmune hepatitis; HUS, acute hemolytic uremic syndrome; CRF, chronic renal failure; LTx, liver transplantation; UC, ulcerative colitis; CD, Crohn’s disease).

Out of a total IBD cohort of 1073 CD patients and 464 UC patients analyzed in this study, 31 patients (2.0% of all IBD patients) underwent SOT during the study period (between July 2002 and May 2014). Among the 31 IBD patients were 22 UC patients (71.0%) and 9 CD patients (29.0%). Therefore, 0.84% of all 1073 CD patients and 22 of all 464 UC patients (4.74%) underwent SOT, confirming the increased incidence of SOT among UC patients compared to CD patients (p = 2.69 x 10^−6^, UC vs. CD). Twenty-four IBD patients underwent LTx (77.4%), six IBD patients underwent kidney transplantation (19.4%) and one patient underwent heart transplantation (3.2%; Table 1, Fig 1).

**Table 1:** Shown are the clinical characteristics of the 31 IBD patients who underwent solid organ transplantation (SOT). Given are sex, age, anti-reject immunosuppressive regimen, malignancies before/after organ transplantation, re-transplantation and reason for re-transplantation and severe complications after SOT, IBD activity before and after SOT, medical treatment of IBD and history of CD-related surgeries. The diagnosis and classification of UC and CD was based on the Montreal classification including endoscopic, radiological, and histopathological parameters [43].

Patient	Age	sex	IBD type	Montreal classi-fication	Indication for SOT	Immuno-suppression after SOT	IBD activity before SOT	IBD activity after SOT	IBD acti-vity change	IBD treatment before SOT	IBD treatment after SOT	surgery before SOT	surgery after SOT	Malignancy before SOT	Malignancy after SOT	Re-Trans-plantation	Reason for Re-Tx	SOT compli-cations	Follow-up months
**IBD patients with liver transplantation (n = 24)**																			
1	53	m	UC	E2	PSC	tac, steroids	remission	mild	worse	5-ASA	no treatment	none	none	none	none	no	n. a.	none	102
2	35	m	UC	E1	PSC	tac	remission	remission	no	5-ASA	no treatment	none	none	none	none	yes(4x LTx)	ischemic Tx organ injury	death because of septic complications /GI bleeding	138
3	46	f	CD	n. a.	PSC	tac	remission	remission	no	AZA	AZA	none	none	none	adeno-carcinoma of the Papilla of Vater	no	n. a.	none	98
4	56	m	UC	E3	PSC	tac, MMF, steroids	severe	remission	better	5-ASA	5-ASA	none	none	none	none	no	n. a.	death because of acute Ischemic Tx organ failure	16
5	44	f	UC	E3	PSC	tac, steroids	remission	severe	worse	5-ASA, steroids	5-ASA, steroids	none	none	none	none	no	n. a.	none	103
6	32	m	UC	E3	PSC	tac	severe	mild	better	AZA	5-ASA, IFX	none	none	none	none	no	n. a.	none	82
7	70	m	UC	E3	PSC	tac, steroids	remission	remission	no	5-ASA, steroids	no treatment	none	none	meso-thelioma	none	no	n. a.	none	141
8	34	f	UC	E3	PSC/AIH overlap	tac, MMF	severe	remission	better	5-ASA, steroids	no treatment	procto-colec-tomy J-pouch	none	none	none	no	n. a.	none	56
9	53	m	CD	L2/B1	PSC	tac, steroids	mild	remission	better	no treatment	no treatment	none	none	none	none	no	n. a.	none	143
10	48	f	UC	E2	PSC	tac, steroids	remission	n. a.	n. a.	no treatment	no treatment	none	none	CCC	none	no	n. a.	none	13
11	32	m	UC	E3	PSC	tac, steroids	remission	mild	worse	5-ASA	5-ASA, ADA	none	none	none	none	yes	ischemic Tx organ injury (split-liver)	none	140
12	59	m	UC	E3	PSC	tac, steroids	mild	mild	no	5-ASA, steroids	no treatment	none	none	HCC	none	no	n. a.	none	127
13	43	m	UC	E3	PSC	tac, steroids	severe	remission	better	5-ASA, steroids	no treatment	procto-colectomy J-pouch (DALM)	none	DALM (procto-colectomy J-pouch)	none	no	n. a.	none	50
14	33	m	UC	E3	PSC/AIH overlap	tac, steroids	mild	remission	better	5-ASA, steroids	no treatment	none	none	none	none	no	n. a.	none	145
15	44	f	UC	E3	PSC	tac, MMF, steroids	mild	mild	no	5-ASA	5-ASA	none	none	none	none	no	n. a.	none	115
16	60	m	UC	E3	PSC	tac, steroids	remission	remission	no	5-ASA	no treatment	none	none	none	none	yes	ischemic Tx organ injury	none	9
17	65	m	UC	E3	PSC	tac	mild	mild	no	5-ASA, steroids	IFX	none	Procto-colec-tomy, J-pouch	none	none	no	n. a.	none	157
18	44	m	UC	E3	PSC/ hemo-chromatosis	tac, steroids	remission	mild	worse	5-ASA, steroids	steroids, AZA	none	none	none	none	yes	chronic Tx organ failur	none	154
19	44	m	CD	L1/B1	PSC	tac, CyA, steroids	remission	remission	no	steroids	no treatment	none	none	CCC	none	no	n. a.	death because of septic complications /bleeding	7
20	53	f	UC	E2	PSC	tac, steroids	remission	remission	no	no treatment	5-ASA	none	none	none	none	no	n. a.	death because of acute ischemic Tx organ failure	41
21	65	m	UC	E2	PSC	tac, steroids	remission	remission	no	5-ASA	5-ASA	none	none	none	none	no	n. a.	death because of acute ischemic Tx organ failure	15
22	44	m	UC	E2	PSC	tac	remission	remission	no	5-ASA	5-ASA	none	none	none	none	no	n. a.	none	139
23	49	f	UC	E3	PSC	tac, steroids	severe	remission	better	no treatment	no treatment	procto-colec-tomy J-pouch	none	none	none	no	n. a.	none	155
24	56	m	UC	E3	PSC	tac	remission	remission	no	no treatment	no treatment	left-sided hemi-colec-tomy, rectal resection for colon cancer	none	sigmoid colon carcinoma	none	no	n. a.	acute cardiac failure	86
**IBD patients with renal transplantation (n = 6)**																			
25	48	f	UC	E3	CRF due to IgA nephropathy	CyA, steroids	remission	mild	worse	no treatment	5-ASA, steroids	none	none	none	none	no	n. a.	none	102
26	63	m	CD	L3/B1	CRF unknown etiology	CyA, MMF, steroids	remission	remission	no	no treatment	no treatment	none	none	none	none	no	n. a.	none	112
27	54	m	CD	n. a.	CRF due to HUS	CyA, steroids	remission	remission	no	no treatment	no treatment	none	none	none	post-transplant lympho-proliferative disorder (PTLD)	yes	ischemic Tx organ injury	none	181
28	45	m	CD	L3/B3p	CRF due to AA amyloidosis	tac, MMF	remission	remission	no	5-ASA, steroids, 6-MP	steroids, 6-MP	ileocecal resection, fistula surgery	none	none	none	no	n. a.	none	108
29	48	m	CD	L3/B3p	CRF due to AA amyloidosis	tac, steroids	remission	mild	no	steroids, AZA	no treatment	ileocecal resection, fistula surgery	none	none	renal cell carcinoma of the Tx kidney	no	n. a.	none	13
30	67	m	CD	L3/B3p	CRF due to oxalate nephropathy	CyA, steroids	remission	remission	no	6-MP	6-MP	ileocecal resection, re-resection of anastomosis-stenosis, fistula surgery	none	none	none	no	n. a.	none	159
**IBD patient with heart transplantation (n = 1)**																			
31	37	m	CD	L2/B2	congestive heart failure because of ischemic dilatative cardio-myopathy	tac, MMF, steroids	mild	remission	better	no treatment	IFX	right-sided hemi-colec-tomy	none	none	none	no	n. a.	none	81

Disease classification was performed as detailed in the Materials and Methods section (n. a., not applicable; DALM, dysplasia-associated lesion or mass; 5-ASA, 5-aminosalicylic acid; AZA, azathioprine; 6-MP, 6-mercaptopurine; ADA, adalimumab; IFX, infliximab; LTx, liver transplantation; Tx, transplantation; PSC, primary sclerosing cholangitis; AIH, autoimmune hepatitis; DALM, Dysplasia-associated lesion or mass; HCC, hepatocellular carcinoma; CCC, cholangio-cellular carcinoma; CRF, chronic renal failure; MMF, mycophenolate mofetil; CyA, cyclosporine A; tac, tacrolimus).

### Outcome of liver transplantation in IBD patients

Twenty-four IBD patients including 21 UC patients and 3 CD patients underwent LTx for PSC or PSC/AIH overlap syndrome; one UC patient had concomitant PSC and hemochromatosis (Fig 1, Table 1). The median age at first diagnosis of liver disease was 27.2 years (range 12.5–56.0 years), compared to a median age of 21.2 years at first IBD diagnosis (range 9.1–50.0 years). The median age at first LTx was 41.2 years (range 27.1–66.0 years). Therefore, the median interval from first diagnosis of liver disease to LTx was 156.8 months (range 10.1–418.0 months).

All patients with LTx received immunosuppressive therapy with tacrolimus after transplantation, five patients received tacrolimus in combination with mycophenolate mofetil (MMF) (20.8% of all LTx patients), 17 patients received concomitant steroid treatment (70.8%) and one UC patient received consecutively cyclosporine A and then tacrolimus (4.2%, Table 1). Three of the 24 IBD patients (12.5%) needed re-transplantation because of acute ischemic organ failure after first LTx; one of them (4.2%) required even a total of four LTx due to recurrent acute ischemic organ failures after transplantation (Table 1). One UC patient (4.2%) needed a second LTx one month after first SOT because of organ failure after recurrent cholangitis and intra-hepatic bleeding complications. Another UC/PSC patient (4.2%) needed re-transplantation three years after first LTx because of chronic vascular complications resulting in a chronic ischemic organ failure (Table 1).

### Outcome of renal transplantation in IBD patients

Six IBD patients out of 1537 IBD patients analyzed (0.4%), including five CD patients (0.47% of all CD patients) and one UC patient (0.22% of all UC patients) underwent renal transplantation for terminal renal failure (Fig 1, Table 1). Two out of these six patients (33%) were diagnosed with AA amyloidosis resulting in chronic renal failure. Another UC patient (16.7%) was diagnosed with IgA nephropathy. One CD patient (16.7%) was diagnosed with oxalate nephropathy and consecutive chronic renal failure needing hemodialysis followed by renal transplantation. Another CD patient (16.6%) developed an acute hemolytic uremic syndrome with acute renal failure and underwent renal transplantation (Table 1). In one CD patient with chronic renal failure, the cause for renal failure could not been diagnosed (Table 1). Median age at first diagnosis of renal disease was 35.8 years (range 14.4–56.9 years) compared to a median age at first diagnosis of IBD of 27.1 years (range 8.4–43.0 years). The median age at first renal transplantation was 43.6 years (range 28.5–66.3 years). Therefore, the median interval from first diagnosis of kidney disease to renal transplantation was 101.0 months (range 0.0–177.1 months). Three of the six IBD patients (50.0%) received cyclosporine A (CyA) after renal transplantation (one patient with CyA mono therapy, one CyA with steroids, and one CyA with MMF treatment, Table 1). Two IBD patients (33.3%) received tacrolimus after kidney transplantation (one patient with combination therapy with steroids and one patient with concomitant MMF treatment, Table 1). Another IBD patient received MMF and steroid treatment after renal transplantation. In summary, five of the six IBD patients (83.3%) had concomitant steroid treatment after transplantation.

### Outcome of solid organ transplantation in the IBD patient with heart transplantation

One CD patient out of 1537 IBD patients analyzed (0.07%) underwent heart transplantation because of ischemic dilatative cardiomyopathy with consecutive congestive heart failure at age of 29 years (Fig 1, Table 1). This patient was diagnosed with CD at age of 22 years and received tacrolimus, MMF and steroid treatment after heart transplantation. The time from diagnosis of heart failure to heart transplantation was 15 months.

### IBD activity and medical treatment before and after solid organ transplantation

Clinical characteristics of UC and CD based on the Montreal classification, disease activity before and after SOT, as well as history of IBD-related surgery and IBD-related medical treatment before and after SOT are given for the 22 UC patients and 9 CD patients in Table 1. All 31 IBD patients underwent endoscopy within a median of 16.5 months before SOT; they underwent also endoscopy within a median of 24.4 months after SOT.

**Fig 2:**
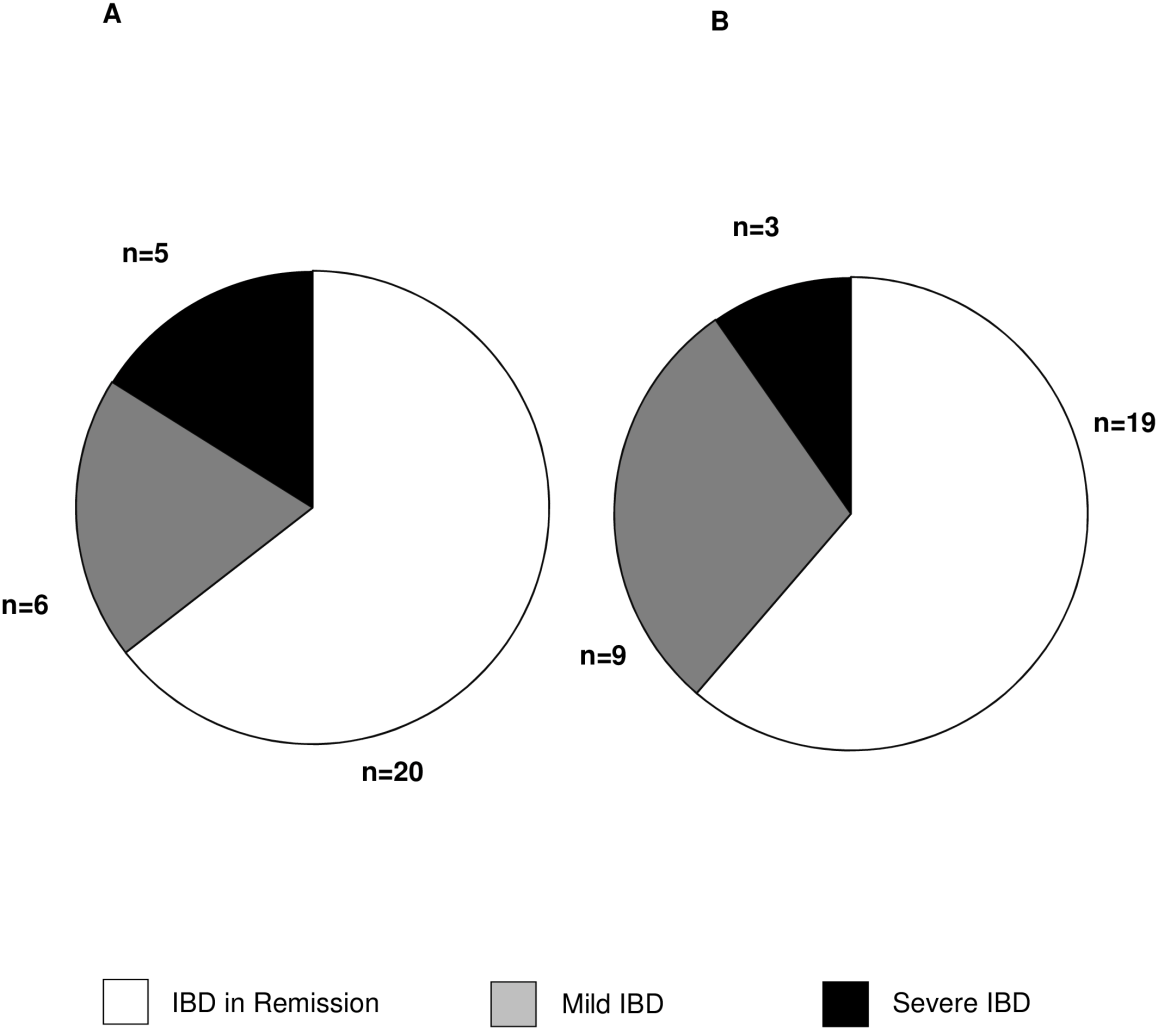
Endoscopic disease activity seen (A) before single organ transplantation (SOT) and (B) after SOT. Overall, 20 of the 31 IBD patients were endoscopically in remission before SOT (64.5%) compared to six IBD patients with mild disease activity before SOT (19.4%) and five IBD patients with severe IBD activity before SOT (16.1%). After SOT, no activity of IBD was endoscopically seen in 19 of the 31 IBD patients (61.3%) versus nine IBD patients with mild disease activity after SOT (29.0%) and severe disease activity in three IBD patients (9.7%).

Overall, 20 of the 31 IBD patients were clinically and endoscopically in remission before SOT (64.5%). Six IBD patients had mild disease activity before SOT (19.4%) and five IBD patients had severe IBD activity before SOT (16.1%, Table 1, Fig 2A). After SOT, no activity of IBD was endoscopically seen in 19 of the 31 IBD patients (61.3%), while nine IBD patients had mild disease activity after SOT (29.0%) and three patients (9.7%) had severe disease activity (Table 1, Fig 2B).

**Fig 3:**
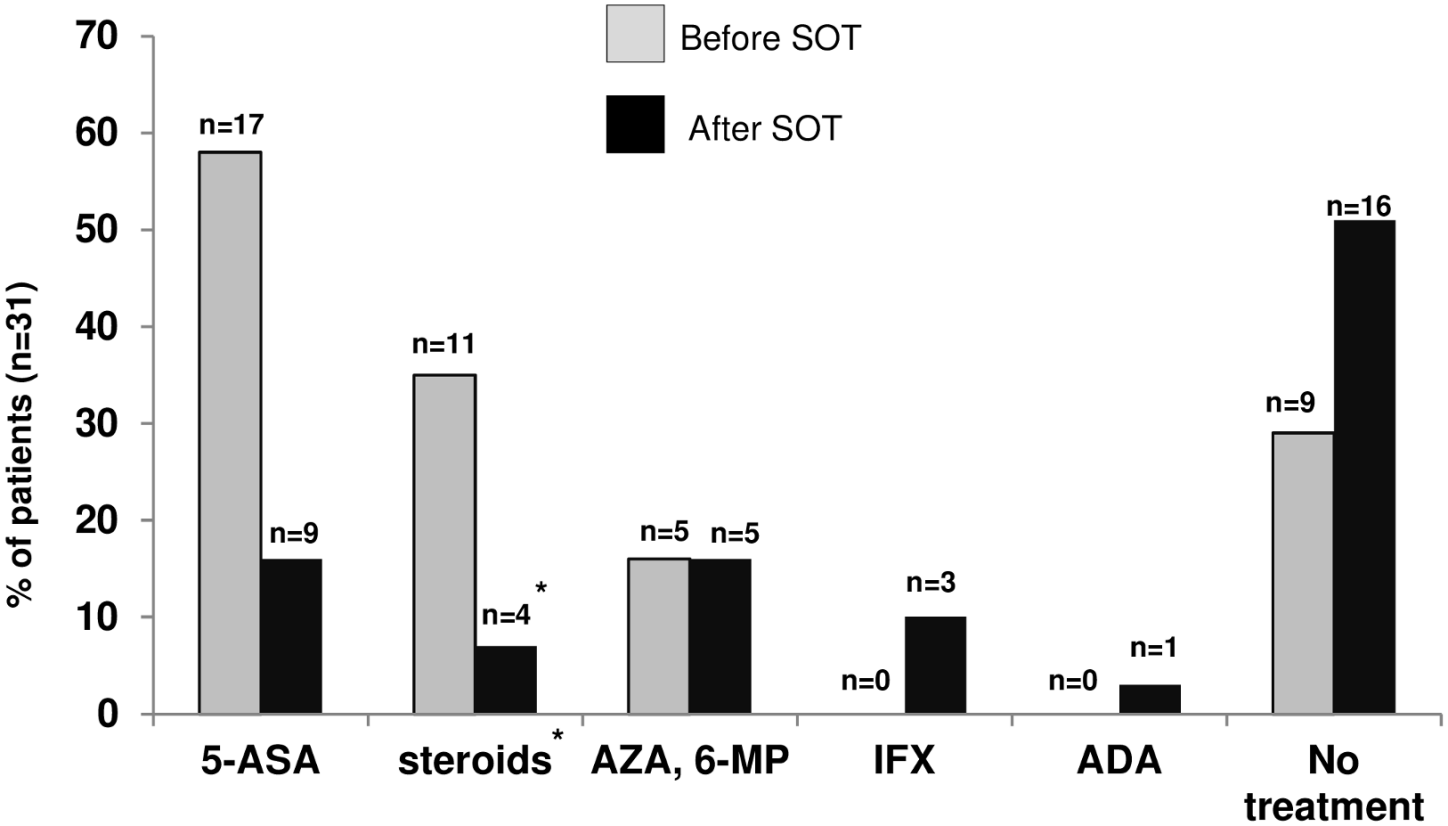
Medical treatment for IBD before and after single organ transplantation (n = 31). Given are the total number of patients and proportion of patients for the relevant IBD treatment. (5-ASA, aminosalicylates; AZA, azathioprine; 6-MP, 6-mercaptopurine; IFX, infliximab; ADA, adalimumab). * excludes short-term steroid treatment for SOT.

History of medical treatment before and after SOT is given in Table 1 and Fig 3. Sixteen UC patients (out of 22 UC patients with SOT; 72.8%) received 5-amino-salicylic acid (5-ASA) treatment pre-SOT, eight patients received steroids (36.4%) and one patient with severe pancolitis received azathioprine (4.5%), while five UC patients had no maintenance treatment before SOT (22.7%, Table 1, Fig 3). After SOT, nine of the 22 UC patients received 5-ASA treatment (40.9%), three patients needed steroid treatment (13.6%) and two UC patients had immunosuppressive therapy with azathioprine after SOT (9.1%, Table 1, Fig 3). Two UC patients (9.1%) were treated with infliximab after SOT and another UC patient was treated with adalimumab 2.5 years after SOT (Table 1, Fig 3).

Four of the nine CD patients had no maintenance treatment before SOT (44.4%, Table 1, Fig 3), two CD patients had immunosuppressive therapy with azathioprine (22.2%) and two other CD patients received 6-mercaptopurine (22.2%). Three CD patients had steroid treatment with two of them receiving concomitant immunosuppressive treatment with azathioprine and 6-mercaptopurine, respectively, and 5-ASA in one of these patients (33.3%, Table 1, Fig 3). After SOT, one CD patient was treated with infliximab (Table 1 and Fig 3). One patient received steroid treatment after SOT because of increased CD activity (11.1%), 3 patients had immunosuppressive therapy with azathioprine after SOT (33.3%) and five patients (55.6%) had no IBD-specific treatment after SOT (Table 1, Fig 3).

### IBD activity changes after solid organ transplantation

**Fig 4:**
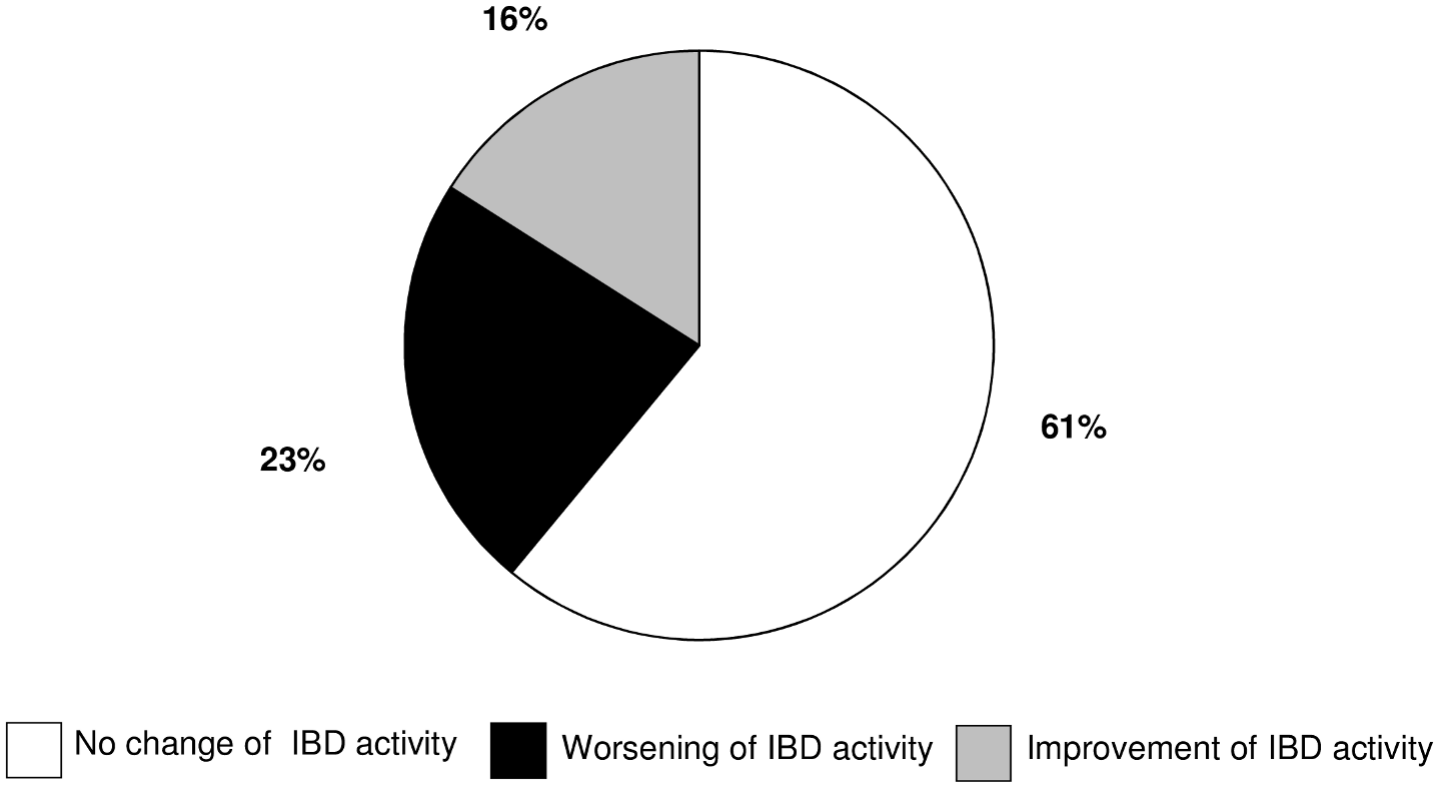
Change of IBD activity in our cohort of 31 IBD patients.

A change of disease activity was seen in twelve of the 31 IBD patients after single organ transplantation (39%), while in 19 patients (61%) no significant influence of SOT on IBD activity was observed. Worsening of disease activity after SOT was seen in 7 patients (23%), while IBD activity decreased in five patients after SOT (16%; Table 1, Fig 4).

Univariate analysis revealed that requirement of additional corticosteroid therapy (defined as prednisolone equivalent of 10 mg or greater for IBD activity and not for therapy of transplant rejection) was a good predictor for worsening IBD activity after SOT (p = 0.03; relative risk (RR): 10.29; 95% CI 1.26–84.06; Table 2).

**Table 2:** Comparison of IBD-SOT patients with unchanged or improved IBD activity (n = 24) and IBD-SOT patients with worsened IBD activity (n = 7; univariate analysis). Steroid treatment for IBD after SOT was significantly associated with worsening of disease activity (p = 0.028). However, this association may be most likely explained by the fact that IBD patients with worsening of IBD activity after SOT will be primarily treated with steroid treatment rather than steroid treatment being an independent risk factor for worsening of IBD activity after SOT.

Variable	Disease activity unchangedor improved after SOT(*n = 24*)	Worsening of diseaseactivity after SOT(*n = 7*)	OR	95% CI	p value
Male sex	18/24 (75.0%)	5/7 (71.4%)	1.193	0.091/10.112	1.000
Age < 20 years at diagnosis of IBD	7/24 (29.2%)	4/7 (57.1%)	0.322	0.037/2.449	0.210
Smoking at date of SOT	1/24 (4.2%)	1/7 (14.3)	0.276	0.003/23.864	0.406
IBD in remission before SOT	13/20 (65.0%)	7/20 (35.0%)	3.337	0.794/15.458	0.113
Diagnosis UC	17/24 (70.8%)	5/7 (71.4%)	0.972	0.076/7.954	1.000
Diagnosis CD	7/24 (29.2%)	2/7 (28.6%)	1.028	0.126/13.243	1.000
Aminosalicylates before SOT	14/24 (58.3%)	3/7 (43.0%)	1.829	0.248/15.396	0.671
Steroid treatment before SOT	8/24 (33.3%)	3/7 (43.0%)	0.676	0.088/5.757	0.676
Azathioprine before SOT	5/24 (20.8%)	1/7 (14.3%)	1.558	0.13/86.917	1.000
Tacrolimus after SOT	22/24 (91.7%)	5/7 (71.4%)	4.135	0.245/70.752	0.212
Cyclosporine A after SOT	2/24 (8.3%)	2/7 (28.6%)	0.242	0.014/4.075	0.212
Aminosalicylates after SOT	6/24 (25.0%)	3/7 (42.9%)	0.457	0.057/4.032	0.384
Azathioprine after SOT	4/24 (16.7%)	1/7 (14.3%)	1.193	0.091/68.793	1.000
Anti-TNF (infliximab or adalimumab) after SOT	3/24 (12.5%)	1/7 (14.3%)	0.862	0.056/52.407	1.000
Steroid treatment for IBD after SOT	1/24 (4.2%)	3/7 (42.9%)	0.058	0.0048/0.7062	***0*.*028***
Mycophenlate mofetil (MMF) after SOT	8/24 (33.3%)	2/7 (28.6%)	1.241	0.155/15.778	1.000

### Severe complications after solid organ transplantation

Overall, there was a survival rate of 83.9% after a mean follow-up period of 33.3 months (range 0.1–242.4 months) after SOT. During a total follow-up time of 103.0 months (range 7.0–182.0 months) and a median follow-up of 33.3 months (range 0.1–242.4 months) after SOT, a total of 6 IBD patients who underwent SOT died (16.1%) at a median age of 49.0 years (range 34.6–57.2 years, Table 1). All patients who died during the follow-up interval were male patients.

Three male UC patients who underwent LTx for PSC (9.7%) died because of acute ischemic failure of the transplanted liver after a median of 5.8 months (range 3.6–8.9 months). One male CD-PSC patient (3.2%) died one year after LTx because of septic and bleeding complications at an age of 35 years. This patient received MMF, steroids and tacrolimus as immunosuppressive regimen after SOT. Another male UC patient who underwent a total of four LTx because of recurrent acute ischemic organ failures died six years after the last LTx because of septic complications and gastro-intestinal bleeding complications at an age of 35 years with concomitant immunosuppressive therapy with tacrolimus (3.2%, Table 1). Two years after LTx, another male UC-PSC patient died because of congestive heart failure (3.2%, Table 1). Despite incomplete data regarding the cytomegalovirus (CMV) infection status before SOT, none of the 31 IBD patients with SOT developed infectious complications related to CMV or CMV reactivation.

### Malignancies in IBD patients before and after SOT

Almost one third of the 31 IBD-SOT patients were diagnosed with malignancies or dysplasia (n = 9, 29%). Six IBD patients (19.4%) were diagnosed with malignancy or dysplasia before SOT (6/9 patients, 66.7%: one male UC patient with abdominal cutaneous malignant mesothelioma; one female UC patient with cholangiocellular carcinoma diagnosed in the explanted liver; one male UC patient with hepatocellular carcinoma diagnosed in the explanted liver; one male UC patient with severe pancolitis was diagnosed with high-grade dysplasia-associated lesions (DALM); one male CD patient with cholangiocellular carcinoma diagnosed in the explanted liver and one UC patient with colorectal cancer before SOT; Table 1).

Three IBD patients (9.7%) were diagnosed with malignancies after SOT (one female UC patient with adenocarcinoma of the papilla of Vater; one male CD patient with post-transplant lymphoproliferative disease (PTLD) and male CD patient with papillary renal cell carcinoma in the transplanted kidney; Table 1).

## Discussion

The aim of this study was to analyze the effect of SOT on the IBD course. Only a minority of 2% of all IBD patients (31 out of 1537 IBD patients) needed SOT in our IBD cohort demonstrating that this is an overall rare event in IBD, especially in CD patients. Importantly, significantly more UC patients underwent SOT in our study cohort compared to CD patients due to the higher prevalence of PSC-related liver cirrhosis in UC (4.74% of all UC patients vs. 0.84% of all CD patients, p = 2.69 x 10^−6^). All LTx were performed due to PSC or PSC overlap syndromes. Epidemiologic data from Northern European countries demonstrated a lifetime risk of 5% for developing PSC in IBD patients [6]. Also in Northern European countries, PSC is a major indication for LTx constituting approximately 17% of all indications for LTx in the general population (including IBD patients) [21].

Overall, outcome of SOT in the 31 patients was favourable in our cohort. The survival rate was 84% (n = 26) during a total follow-up of 103.0 months (range 7.0–182.0 months) and a median follow-up period of 33.3 months after SOT (Table 1). Five male IBD patients who underwent SOT died (16%) at a median age of 49.0 years. Most common complications were ischemic organ failure of the transplanted liver, septic complications as well as uncontrollable bleeding complications.

Renal failure is a rare complication especially in patients with CD [20]. Age and duration of IBD have been identified as independent risk factors to develop renal failure [44]. Systemic AA amyloidosis is associated with IBD and at least 1% of IBD patients will develop amyloidosis [45]. Two of our CD patients needed renal transplantation for AA amyloidosis and had favourable long-term outcomes. An association between IgA nephropathy and IBD seems possible [46] and there is an between oxalate nephropathy and IBD since the prevalence of calcium-oxalate urolithiasis is up to five-fold higher in CD than in the general population [47]. Hemolytic-uremic syndrome (HUS) is characterized by microangiopathic hemolytic anemia, impaired renal function and excessive platelet consumption leading to thrombocytopenia especially related to gastrointestinal tract infections with Shiga toxin-producing *Escherichia coli* (STEC) [48]. CD seems to be a likely predisposing factor for HUS because of recurrent gastrointestinal tract infections [49].

Importantly, the majority of IBD patients in our cohort received a tacrolimus-based anti-reject treatment regimen after SOT (87.1%). In some studies, this immunosuppressive treatment regimen was associated with an unfavourable outcome in IBD patients who underwent SOT with an up to four-fold higher risk of post-LTx IBD relapse [5, 50–52]. However, we could not confirm this unfavourable outcome in IBD patients with tacrolimus-based anti-reject treatment regimen post-SOT as in 61% of patients disease activity was not influenced by SOT (and SOT-associated immunosuppressive therapy) and 16% of patients had even improvement of disease activity after SOT.

Cyclosporine-based anti-reject regimens after SOT were not associated with worsening of disease activity in patients with IBD [52, 53]. However, only four of our 31 IBD patients (13.0%, 3 CD patients and one UC patient) had cyclosporine-based immunosuppression after SOT (Table 1). Disease activity did not change in two of these patients after start of cyclosporine A; two patients had mild activity after SOT while all patients were clinically and endoscopically in remission before SOT. However, our subgroup of patients with cyclosporine A treatment after SOT is too small to draw definite conclusions. These observations were confirmed by univariate analysis of risk factors, demonstrating no association between tacrolimus or cyclosporine treatment after SOT with worsening of disease activity.

Steroid treatment for IBD after SOT was associated with active disease in this univariate analysis (p = 0.028, Table 2). This association may be most likely explained by the fact that patients with active IBD after SOT will be primarily started with steroid treatment to control disease activity considering the limited experience with other treatment options for IBD maintenance therapy after SOT such as anti-TNF treatment. Therefore, steroid therapy is not necessarily a predictor of disease worsening after SOT but rather an indicator for active IBD following SOT.

Based on the results of a large Scandinavian meta-analysis with unfavourable outcomes of IBD under tacrolimus-based anti-reject treatment regimen after liver transplantation, Jørgensen et al. suggested a shift of immunosuppressive treatment to cyclosporine as potentially beneficial [5, 21]. However, tacrolimus-based anti-reject therapy seems superior to cyclosporine-based anti-reject treatment regimen by significantly reducing the risk of acute rejection and steroid-resistant rejection as well as the risk of graft loss [54]. For every 100 LTx patients treated with tacrolimus instead of cyclosporine, rejection and graft loss could be avoided in 9 and 5 patients, respectively [54]. None of the IBD patients in our cohort had severe episodes of acute rejection after SOT or loss of the transplant due to acute rejection reaction. Therefore, our data cannot support unfavourable outcomes of the IBD course in tacrolimus-treated patients. Taking the lower risk of acute rejection and steroid-resistant rejection as well as the lower risk of graft loss in patients with tacrolimus treatment into account, a switch to cyclosporine in IBD patients with SOT cannot be recommended considering the results of our study.

Although calcineurin inhibitors (CNIs) are the main anti-reject treatment after LTx, CNI treatment is associated with unfavourable side effects such as worsening of renal dysfunction, neurotoxicity, and diabetes in patients following LTx. The use of mammalian target of rapamycin (mTOR) inhibitors after liver transplantation has been associated with favourable benefits on renal function but with efficacy comparable to CNIs and therefore would be a good alternative in IBD patients following LTx [55]. However, data on mTOR treatment for IBD are very limited and currently not established to control disease activity in patients with IBD [55].

Data on the prevalence of colectomy after SOT are conflicting. Whereas a progressive PSC with a consecutive need for LTx seems to be associated with a decrease of disease activity in some IBD/PSC patients, other clinical trials report a prevalence of colectomy of up to 35% in UC patients after LTx [56, 57]. In our cohort, only one patient needed colectomy after SOT because of refractory pancolitis despite anti-TNF maintenance treatment with infliximab.

In the literature, a total of 21 patients with anti-TNF treatment after SOT are reported to date [36–40]. The majority of these patients showed good response rates after start of anti-TNF treatment. With the exception of one study [37], which demonstrated in several patients infectious complications and a case of post-transplant lymphoproliferative disorder, there was also an overall good safety outcome (Table 3). Considering the patient number of these studies combined (n = 21), the clinical experience of anti-TNF-treated IBD patients with SOT is still very limited. In addition, the overall incidence of SOT in IBD is rare; therefore, very large studies are needed to draw definitive conclusions on the safety of anti-TNF therapies in SOT patients.

**Table 3:** Given is an overview of publications on IBD patients who received anti-TNF therapy after solid organ transplantation including the 4 anti-TNF-treated patients of this study.

Author	Number of patients treated with anti-TNF therapy	Anti-TNF treatment (IFX, ADA)	Clinical outcome, Response rate (%)	Endoscopic outcome, mucosal healing(%)	Adverse events
Sandhu et al. [36]	6	6 patients with IFX	67	n/a	Systemic lupus erythematosus, Colorectal cancer
Mohabbat et al. [37]	8	4 patients with IFX 2 patients with ADA after IFX, 2 patients with ADA	87.5	42.9	Oral candidiasis, Clostridium difficile colitis, Bacterial pneumonia, Cryptosporidiosis,Epstein-Barr virus-positive post-transplant lympho-proliferative disorder
Lal et al. [38]	1	1 patient with IFX	100	100	None
El-Nachef et al. [39]	2	1 patient with IFX1 patient with ADA	100	n/a	None
Indriolo et al. [40]	4	4 patients with IFX	75	33	Molluscum contagiosum
Schnitzler et al. (Own data)	4	3 patients with IFX, one patient with ADA	75	75	None

A total of 21 IBD patients received anti-TNF therapy after SOT, including 17 patients who received IFX and 4 IBD patients who received ADA treatment after organ transplantation (n/a, not applicable; IFX, infliximab; ADA, adalimumab).

In our cohort, four patients (13.0%) received anti-TNF treatment after SOT, including one CD patient after heart transplantation. This patient suffered from an inflammatory intestinal stenosis before heart transplantation. After start of infliximab, this patient was clinically in remission; endoscopically no signs of inflammation were seen. No side effects occurred. This is to our knowledge the first report of an IBD patient with infliximab treatment after heart transplantation. Overall, outcome of anti-TNF treatment was good in our cohort, although the number of patients is small. None of the four anti-TNF treated patients developed infectious complications; in one UC patient infliximab treatment was stopped prophylactically because of recurrent episodes of cholangitis most likely caused by stenosis of the biliary-enteric anastomosis. Despite the limited data on anti-TNF therapy, anti-TNF treatment seems effective and safe in IBD patients post-SOT and refractory to conventional treatment [36–40]. Only one UC patient needed surgery after SOT with proctocolectomy and ileal pouch-anal anastomosis for treatment-refractory UC. Tacrolimus-based reject therapy after solid organ transplantation had a favourable outcome in patients with IBD. The risk for colorectal cancer was low in our IBD cohort, only one UC patient (3%) was diagnosed with colorectal cancer before SOT and none of the IBD patients were diagnosed with colorectal cancer after SOT. However, given that the majority of patients were PSC-IBD patients with a high risk for developing colorectal cancer, annual screening colonoscopies were performed in most patients, likely contributing to the low number of colorectal cancers.

Our study represents one of the largest single-center experiences on SOT outcomes in IBD patients. A major limitation of our study was the limited number of patients included in the analysis; e.g., the subgroup of anti-TNF-treated patients included only four patients. However, considering that the total number of all anti-TNF-treated IBD-SOT patients in the medical literature is only n = 21, this study adds important information to our knowledge of how to treat IBD patients after SOT.

In conclusion, due to the stronger association of PSC-associated liver cirrhosis with UC (compared to CD), SOT is significantly more often required in UC (4.74% of our patients) than in CD (0.84% of our patients; p = 2.69 x 10^−6^, UC vs. CD). The overall outcome of SOT in our IBD cohort was favourable with a survival rate of 84%. Anti-TNF treatment was effective and safe in all IBD patients who underwent SOT. This suggests good safety aspects of anti-TNF-treatment in IBD after SOT, although larger, multi-center cohort analyses are needed to confirm these findings.

## References

[1] Gizard E, Ford AC, Bronowicki JP, Peyrin-Biroulet L. Systematic review: The epidemiology of the hepatobiliary manifestations in patients with inflammatory bowel disease. Alimentary pharmacology & therapeutics. 2014;40(1):3–15. . 24815622 10.1111/apt.12794

[2] Oikonomou K, Kapsoritakis A, Eleftheriadis T, Stefanidis I, Potamianos S. Renal manifestations and complications of inflammatory bowel disease. Inflammatory bowel diseases. 2011;17(4):1034–45. . 20842645 10.1002/ibd.21468

[3] Rust C, Brand S. PSC: Protect and serve with colitis: does it help the liver to have severe ulcerative colitis? Gut. 2011;60(9):1165–6. 10.1136/gut.2011.240309 . 21561875

[4] Eaton JE, Talwalkar JA, Lazaridis KN, Gores GJ, Lindor KD. Pathogenesis of primary sclerosing cholangitis and advances in diagnosis and management. Gastroenterology. 2013;145(3):521–36. 23827861 10.1053/j.gastro.2013.06.052 PMC3815445

[5] Singh S, Loftus EV Jr., Talwalkar JA. Inflammatory bowel disease after liver transplantation for primary sclerosing cholangitis. The American journal of gastroenterology. 2013;108(9):1417–25. 10.1038/ajg.2013.163 . 23896954

[6] Olsson R, Danielsson A, Jarnerot G, Lindstrom E, Loof L, Rolny P, et al Prevalence of primary sclerosing cholangitis in patients with ulcerative colitis. Gastroenterology. 1991;100(5 Pt 1):1319–23. . 2013375

[7] Broome U, Bergquist A. Primary sclerosing cholangitis, inflammatory bowel disease, and colon cancer. Seminars in liver disease. 2006;26(1):31–41. 10.1055/s-2006-933561 . 16496231

[8] Yanai H, Matalon S, Rosenblatt A, Awadie H, Berdichevski T, Snir Y, et al Prognosis of Primary Sclerosing Cholangitis in Israel is Independent of Coexisting Inflammatory Bowel Disease. Journal of Crohn's & colitis. 2014 10.1093/ecco-jcc/jju013 . 25518055

[9] Loftus EV Jr., Harewood GC, Loftus CG, Tremaine WJ, Harmsen WS, Zinsmeister AR, et al PSC-IBD: a unique form of inflammatory bowel disease associated with primary sclerosing cholangitis. Gut. 2005;54(1):91–6. 10.1136/gut.2004.046615 15591511 PMC1774346

[10] Boonstra K, van Erpecum KJ, van Nieuwkerk KM, Drenth JP, Poen AC, Witteman BJ, et al Primary sclerosing cholangitis is associated with a distinct phenotype of inflammatory bowel disease. Inflammatory bowel diseases. 2012;18(12):2270–6. . 22407885 10.1002/ibd.22938

[11] Joo M, Abreu-e-Lima P, Farraye F, Smith T, Swaroop P, Gardner L, et al Pathologic features of ulcerative colitis in patients with primary sclerosing cholangitis: a case-control study. The American journal of surgical pathology. 2009;33(6):854–62. . 19295408 10.1097/PAS.0b013e318196d018

[12] O'Toole A, Alakkari A, Keegan D, Doherty G, Mulcahy H, O'Donoghue D. Primary sclerosing cholangitis and disease distribution in inflammatory bowel disease. Clinical gastroenterology and hepatology: the official clinical practice journal of the American Gastroenterological Association. 2012;10(4):439–41. . 22094024 10.1016/j.cgh.2011.11.010

[13] Sokol H, Cosnes J, Chazouilleres O, Beaugerie L, Tiret E, Poupon R, et al Disease activity and cancer risk in inflammatory bowel disease associated with primary sclerosing cholangitis. World journal of gastroenterology: WJG. 2008;14(22):3497–503. 18567077 10.3748/wjg.14.3497 PMC2716611

[14] Jorgensen KK, Grzyb K, Lundin KE, Clausen OP, Aamodt G, Schrumpf E, et al Inflammatory bowel disease in patients with primary sclerosing cholangitis: clinical characterization in liver transplanted and nontransplanted patients. Inflammatory bowel diseases. 2012;18(3):536–45. . 21456044 10.1002/ibd.21699

[15] Lundqvist K, Broome U. Differences in colonic disease activity in patients with ulcerative colitis with and without primary sclerosing cholangitis: a case control study. Diseases of the colon and rectum. 1997;40(4):451–6. . 9106695 10.1007/BF02258391

[16] Soetikno RM, Lin OS, Heidenreich PA, Young HS, Blackstone MO. Increased risk of colorectal neoplasia in patients with primary sclerosing cholangitis and ulcerative colitis: a meta-analysis. Gastrointestinal endoscopy. 2002;56(1):48–54. . 12085034 10.1067/mge.2002.125367

[17] Broome U, Lofberg R, Veress B, Eriksson LS. Primary sclerosing cholangitis and ulcerative colitis: evidence for increased neoplastic potential. Hepatology. 1995;22(5):1404–8. . 7590655 10.1002/hep.1840220511

[18] Sano H, Nakazawa T, Ando T, Hayashi K, Naitoh I, Okumura F, et al Clinical characteristics of inflammatory bowel disease associated with primary sclerosing cholangitis. Journal of hepato-biliary-pancreatic sciences. 2011;18(2):154–61. . 20740366 10.1007/s00534-010-0319-8

[19] Bergquist A, Ekbom A, Olsson R, Kornfeldt D, Loof L, Danielsson A, et al Hepatic and extrahepatic malignancies in primary sclerosing cholangitis. Journal of hepatology. 2002;36(3):321–7. . 11867174 10.1016/s0168-8278(01)00288-4

[20] Primas C, Novacek G, Schweiger K, Mayer A, Eser A, Papay P, et al Renal insufficiency in IBD—prevalence and possible pathogenetic aspects. Journal of Crohn's & colitis. 2013;7(12):e630–4. 10.1016/j.crohns.2013.05.001 . 23706934

[21] Jorgensen KK, Lindstrom L, Cvancarova M, Karlsen TH, Castedal M, Friman S, et al Immunosuppression after liver transplantation for primary sclerosing cholangitis influences activity of inflammatory bowel disease. Clinical gastroenterology and hepatology: the official clinical practice journal of the American Gastroenterological Association. 2013;11(5):517–23. 10.1016/j.cgh.2012.12.027 . 23333218

[22] Gavaler JS, Delemos B, Belle SH, Heyl AE, Tarter RE, Starzl TE, et al Ulcerative colitis disease activity as subjectively assessed by patient-completed questionnaires following orthotopic liver transplantation for sclerosing cholangitis. Digestive diseases and sciences. 1991;36(3):321–8. 1995269 10.1007/BF01318204 PMC2991115

[23] Joshi D, Bjarnason I, Belgaumkar A, O'Grady J, Suddle A, Heneghan MA, et al The impact of inflammatory bowel disease post-liver transplantation for primary sclerosing cholangitis. Liver international: official journal of the International Association for the Study of the Liver. 2013;33(1):53–61. . 22103794 10.1111/j.1478-3231.2011.02677.x

[24] Befeler AS, Lissoos TW, Schiano TD, Conjeevaram H, Dasgupta KA, Millis JM, et al Clinical course and management of inflammatory bowel disease after liver transplantation. Transplantation. 1998;65(3):393–6. . 9484758 10.1097/00007890-199802150-00017

[25] MacLean AR, Lilly L, Cohen Z, O'Connor B, McLeod RS. Outcome of patients undergoing liver transplantation for primary sclerosing cholangitis. Diseases of the colon and rectum. 2003;46(8):1124–8. . 12907911 10.1007/s10350-004-7291-9

[26] Moncrief KJ, Savu A, Ma MM, Bain VG, Wong WW, Tandon P. The natural history of inflammatory bowel disease and primary sclerosing cholangitis after liver transplantation—a single-centre experience. Canadian journal of gastroenterology = Journal canadien de gastroenterologie. 2010;24(1):40–6. 20186355 10.1155/2010/830291 PMC2830633

[27] Papatheodoridis GV, Hamilton M, Mistry PK, Davidson B, Rolles K, Burroughs AK. Ulcerative colitis has an aggressive course after orthotopic liver transplantation for primary sclerosing cholangitis. Gut. 1998;43(5):639–44. 9824344 10.1136/gut.43.5.639 PMC1727300

[28] Saldeen K, Friman S, Olausson M, Olsson R. Follow-up after liver transplantation for primary sclerosing cholangitis: effects on survival, quality of life, and colitis. Scandinavian journal of gastroenterology. 1999;34(5):535–40. . 10423073 10.1080/003655299750026308

[29] Shaked A, Colonna JO, Goldstein L, Busuttil RW. The interrelation between sclerosing cholangitis and ulcerative colitis in patients undergoing liver transplantation. Annals of surgery. 1992;215(6):598–603; discussion 4–5. 1632681 10.1097/00000658-199206000-00006 PMC1242511

[30] Stephens J, Goldstein R, Crippin J, Husberg B, Holman M, Gonwa TA, et al Effects of orthotopic liver transplantation and immunosuppression on inflammatory bowel disease in primary sclerosing cholangitis patients. Transplantation proceedings. 1993;25(1 Pt 2):1122–3. . 7680144

[31] van de Vrie W, de Man RA, van Buuren HR, Schouten WR, Tilanus HW, Metselaar HJ. Inflammatory bowel disease and liver transplantation for primary sclerosing cholangitis. European journal of gastroenterology & hepatology. 2003;15(6):657–63. . 12840678 10.1097/00042737-200306000-00013

[32] Gheith O, Al-Otaibi T, Tawab KA, Said T, Balaha MA, Halim MA, et al Erythema nodosum in renal transplant recipients: multiple cases and review of literature. Transplant infectious disease: an official journal of the Transplantation Society. 2010;12(2):164–8. . 20002354 10.1111/j.1399-3062.2009.00474.x

[33] Temme J, Koziolek M, Bramlage C, Schaefer IM, Fuzesi L, Ramadori G, et al Infliximab as therapeutic option in steroid-refractory ulcerative colitis after kidney transplantation: case report. Transplantation proceedings. 2010;42(9):3880–2. . 21094876 10.1016/j.transproceed.2010.08.044

[34] Parameswaran S, Singh K, Nada R, Rathi M, Kohli H, Jha V, et al Ulcerative colitis after renal transplantation: A case report and review of literature. Indian journal of nephrology. 2011;21(2):120–2. 10.4103/0971-4065.78063 21769176 PMC3132332

[35] Azevedo P, Freitas C, Aguiar P, Silva H, Santos T, Farrajota P, et al A case series of de novo inflammatory bowel disease after kidney transplantation. Transplantation proceedings. 2013;45(3):1084–7. 10.1016/j.transproceed.2013.03.008 . 23622632

[36] Sandhu A, Alameel T, Dale CH, Levstik M, Chande N. The safety and efficacy of antitumour necrosis factor-alpha therapy for inflammatory bowel disease in patients post liver transplantation: a case series. Alimentary pharmacology & therapeutics. 2012;36(2):159–65. . 22616981 10.1111/j.1365-2036.2012.05141.x

[37] Mohabbat AB, Sandborn WJ, Loftus EV Jr., Wiesner RH, Bruining DH. Anti-tumour necrosis factor treatment of inflammatory bowel disease in liver transplant recipients. Alimentary pharmacology & therapeutics. 2012;36(6):569–74. . 22779779 10.1111/j.1365-2036.2012.05217.x

[38] Lal S, Steinhart AH. Infliximab for ulcerative colitis following liver transplantation. European journal of gastroenterology & hepatology. 2007;19(3):277–80. . 17301656 10.1097/MEG.0b013e3280116ccc

[39] El-Nachef N, Terdiman J, Mahadevan U. Anti-tumor necrosis factor therapy for inflammatory bowel disease in the setting of immunosuppression for solid organ transplantation. The American journal of gastroenterology. 2010;105(5):1210–1. 10.1038/ajg.2010.33 . 20445523

[40] Indriolo A, Fagiuoli S, Pasulo L, Fiorino G, Danese S, Ravelli P. Letter: infliximab therapy in inflammatory bowel disease patients after liver transplantation. Alimentary pharmacology & therapeutics. 2013;37(8):840–2. . 23496317 10.1111/apt.12256

[41] Ha C, Magowan S, Accortt NA, Chen J, Stone CD. Risk of arterial thrombotic events in inflammatory bowel disease. The American journal of gastroenterology. 2009;104(6):1445–51. 10.1038/ajg.2009.81 . 19491858

[42] Indriolo A, Ravelli P. Clinical management of inflammatory bowel disease in the organ recipient. World journal of gastroenterology: WJG. 2014;20(13):3525–33. 10.3748/wjg.v20.i13.3525 24707135 PMC3974519

[43] Silverberg MS, Satsangi J, Ahmad T, Arnott ID, Bernstein CN, Brant SR, et al Toward an integrated clinical, molecular and serological classification of inflammatory bowel disease: report of a Working Party of the 2005 Montreal World Congress of Gastroenterology. Canadian journal of gastroenterology = Journal canadien de gastroenterologie. 2005;19 Suppl A:5A–36A. . 16151544 10.1155/2005/269076

[44] Lewis B, Mukewar S, Lopez R, Brzezinski A, Hall P, Shen B. Frequency and risk factors of renal insufficiency in inflammatory bowel disease inpatients. Inflammatory bowel diseases. 2013;19(9):1846–51. . 23689806 10.1097/MIB.0b013e31828a661e

[45] Sattianayagam PT, Gillmore JD, Pinney JH, Gibbs SD, Wechalekar AD, Gilbertson JA, et al Inflammatory bowel disease and systemic AA amyloidosis. Digestive diseases and sciences. 2013;58(6):1689–97. 10.1007/s10620-012-2549-x . 23371008

[46] Ambruzs JM, Walker PD, Larsen CP. The histopathologic spectrum of kidney biopsies in patients with inflammatory bowel disease. Clinical journal of the American Society of Nephrology: CJASN. 2014;9(2):265–70. 10.2215/CJN.04660513 24262508 PMC3913236

[47] Hueppelshaeuser R, von Unruh GE, Habbig S, Beck BB, Buderus S, Hesse A, et al Enteric hyperoxaluria, recurrent urolithiasis, and systemic oxalosis in patients with Crohn's disease. Pediatric nephrology. 2012;27(7):1103–9. 10.1007/s00467-012-2126-8 . 22366809

[48] Kaplan BS, Meyers KE, Schulman SL. The pathogenesis and treatment of hemolytic uremic syndrome. Journal of the American Society of Nephrology: JASN. 1998;9(6):1126–33. . 9621299 10.1681/ASN.V961126

[49] Peraldi MN, Akposso K, Haymann JP, Lahlou A, Sraer JD. Haemolytic-uraemic syndrome in patients with Crohn's disease. Nephrology, dialysis, transplantation: official publication of the European Dialysis and Transplant Association—European Renal Association. 1997;12(12):2744–5. . 9430887 10.1093/ndt/12.12.2744

[50] Dvorchik I, Subotin M, Demetris AJ, Fung JJ, Starzl TE, Wieand S, et al Effect of liver transplantation on inflammatory bowel disease in patients with primary sclerosing cholangitis. Hepatology. 2002;35(2):380–4. 11826412 10.1053/jhep.2002.30695 PMC2965629

[51] Haagsma EB, Van Den Berg AP, Kleibeuker JH, Slooff MJ, Dijkstra G. Inflammatory bowel disease after liver transplantation: the effect of different immunosuppressive regimens. Alimentary pharmacology & therapeutics. 2003;18(1):33–44. . 12848624 10.1046/j.1365-2036.2003.01613.x

[52] Verdonk RC, Dijkstra G, Haagsma EB, Shostrom VK, Van den Berg AP, Kleibeuker JH, et al Inflammatory bowel disease after liver transplantation: risk factors for recurrence and de novo disease. American journal of transplantation: official journal of the American Society of Transplantation and the American Society of Transplant Surgeons. 2006;6(6):1422–9. . 16686766 10.1111/j.1600-6143.2006.01333.x

[53] Cholongitas E, Papatheodoridis GV, Zappoli P, Giannakopoulos A, Patch D, Marelli L, et al Combined HLA-DR and-DQ disparity is associated with a stable course of ulcerative colitis after liver transplantation for primary sclerosing cholangitis. Liver transplantation: official publication of the American Association for the Study of Liver Diseases and the International Liver Transplantation Society. 2007;13(4):552–7. . 17394153 10.1002/lt.21077

[54] McAlister VC, Haddad E, Renouf E, Malthaner RA, Kjaer MS, Gluud LL. Cyclosporin versus tacrolimus as primary immunosuppressant after liver transplantation: a meta-analysis. American journal of transplantation: official journal of the American Society of Transplantation and the American Society of Transplant Surgeons. 2006;6(7):1578–85. . 16827858 10.1111/j.1600-6143.2006.01360.x

[55] Reinisch W, Panes J, Lemann M, Schreiber S, Feagan B, Schmidt S, et al A multicenter, randomized, double-blind trial of everolimus versus azathioprine and placebo to maintain steroid-induced remission in patients with moderate-to-severe active Crohn's disease. The American journal of gastroenterology. 2008;103(9):2284–92. 10.1111/j.1572-0241.2008.02024.x . 18671816

[56] Navaneethan U, Choudhary M, Venkatesh PG, Lashner BA, Remzi FH, Shen B, et al The effects of liver transplantation on the clinical course of colitis in ulcerative colitis patients with primary sclerosing cholangitis. Alimentary pharmacology & therapeutics. 2012;35(9):1054–63. . 22428731 10.1111/j.1365-2036.2012.05067.x

[57] Ho GT, Seddon AJ, Therapondos G, Satsangi J, Hayes PC. The clinical course of ulcerative colitis after orthotopic liver transplantation for primary sclerosing cholangitis: further appraisal of immunosuppression post transplantation. European journal of gastroenterology & hepatology. 2005;17(12):1379–85. . 16292093 10.1097/00042737-200512000-00018

